# Editorial: Overcoming the cell membrane to target intracellular oncoproteins

**DOI:** 10.3389/fonc.2023.1222095

**Published:** 2023-07-12

**Authors:** Christopher J. Brown, Yong Li, Kanaga Sabapathy

**Affiliations:** ^1^Institute of Molecular and Cell Biology (ASTAR), Singapore, Singapore; ^2^Department of Medicine Baylor College of Medicine, Houston, TX, United States; ^3^Division of Cellular & Molecular Research, Humphrey Oei Institute of Cancer Research, National Cancer Centre Singapore, Singapore, Singapore

**Keywords:** membrane, oncoproteins, macromolecules, permeability, delivery, MYC, p53

Many intracellular macromolecules and their interfaces exist in human cancers that are highly desirable as anti-cancer therapeutic targets, such as Myc:Max, KRAS:RAF, and p53. Some of these interactions present significant hurdles to small molecule development due to the nature of their molecular surfaces, which are much more amenable to alternative therapeutic modalities such as antibodies, macrocyclic peptides, and RNA/DNA aptamers. However, their intracellular location is a major challenge for drug development and delivery as most therapeutics, either small molecules or biologics, are impeded by the cellular membrane, a fluidic barrier that has evolved to carefully regulate the transfer of molecules into and out of the cell. Over the last decade, extensive research efforts have been led to further understand how these emerging modalities can be modified, encoded, encapsulated, and delivered into mammalian cells specifically to engage intracellular targets. The recent success of mRNA vaccines against SAR-CoV-2 has marked a milestone in the development of new molecule-delivery systems, whilst many molecules that are much larger than conventional drugs are achieving cell permeability and oral availability and demonstrate the potential of new modalities to engage intracellular targets.

This Research Topic aims to address the challenges of engaging intracellular targets and macromolecular surfaces by describing recent developments in strategies to engage oncogenes that produce intracellular targets. Weber and Hartl discuss the role of MYC as a major oncogenic driver in most human cancers and the potential strategies to intervene therapeutically. They also summarize current possibilities to deliver appropriate drugs into cancer cells containing derailed MYC activation using viral vectors or appropriate nanoparticles. Further to the approaches described to target MYC, Bonilla et al. demonstrate the use of ultra-large virtual screening libraries of compounds that can be made on demand to identify small molecules against the N-terminal domain of the promising cancer target intracellular STAT3. This protein domain is devoid of significant clefts and/or cavities and considered intractable to small molecule inhibition. Their results suggest that the use of ultra-large virtual compound databases to sample more diverse regions of chemical space can lead to the successful development of small molecule drugs for hard-to-target intracellular proteins. In addition to the description of strategies to target MYC and STAT3, Li et al. review the important role of Yin Yang 1 (YY1) in tumorigenesis and tumor progression and highlight the need to target this transcription factor, for which no specific inhibitor or targeted drug has been identified in the clinic. Additionally, as part of this review topic, Yong et al. report a novel function of p53 as a negative regulator of cisplatin resistance in osteosarcoma. Cisplatin is a first-line chemotherapy drug in osteosarcoma patients, whose efficacy is severely restricted by innate patient or acquired resistance. This article demonstrates that p53 overexpression restricts nuclear translocation of transcription factor specificity protein 1 (SP1) and decreases expression of CTR1, the major influx transporter of cisplatin into cells, and concludes that the p53-SP1-CTR1 axis represents a potential therapeutic strategy for overcoming cisplatin resistance.

The articles presented here highlight a set of provocative intracellular targets that require therapeutic intervention alongside a review of techniques and strategies to identify and deliver modalities to beneficially modulate their function. The gained knowledge will become increasingly valuable as membrane-disruption and carrier-based technologies continue to expand the frontiers of intracellular macromolecule delivery.

## Author contributions

All authors listed have made a substantial, direct, and intellectual contribution to the work and approved it for publication.

